# Contact Investigation of Children Exposed to Tuberculosis in South East Asia: A Systematic Review

**DOI:** 10.1155/2012/301808

**Published:** 2011-11-15

**Authors:** Rina Triasih, Merrin Rutherford, Trisasi Lestari, Adi Utarini, Colin F. Robertson, Stephen M. Graham

**Affiliations:** ^1^Department of Paediatrics, Faculty of Medicine, Gadjah Mada University, Yogyakarta 55284, Indonesia; ^2^Centre for International Child Health, Department of Paediatrics and Murdoch Childrens Research Institute, Royal Children's Hospital, University of Melbourne, Melbourne, VIC 3052, Australia; ^3^Department of Respiratory Medicine, Royal Children's Hospital, Melbourne, VIC 3052, Australia; ^4^Center for International Health, University of Otago, Dunedin 9050, New Zealand; ^5^Department of Public Health, Faculty of Medicine, Gadjah Mada University, Yogyakarta 55281, Indonesia; ^6^Child Lung Health, International Union Against Tuberculosis and Lung Disease, 75006 Paris, France

## Abstract

*Background*. Screening of children who are household contacts of tuberculosis (TB) cases is universally recommended but rarely implemented in TB endemic setting. This paper aims to summarise published data of the prevalence of TB infection and disease among child contacts in South East Asia. *Methods*. Search strategies were developed to identify all published studies from South East Asia of household contact investigation that included children (0–15 years). *Results*. Eleven studies were eligible for review. There was heterogeneity across the studies. TB infection was common among child contacts under 15 years of age (24.4–69.2%) and was higher than the prevalence of TB disease, which varied from 3.3% to 5.5%. *Conclusion*. TB infection is common among children that are household contacts of TB cases in South East Asia. Novel approaches to child contact screening and management that improve implementation in South East Asia need to be further evaluated.

## 1. Introduction

South East Asia is home to one-third of the global burden of tuberculosis (TB), with an estimated 5 million prevalent cases and an annual incidence of 3 million TB cases. Five of the 11 countries in the region are among the 22 high-burden countries, with India accounting for over 20% of the global burden of TB disease [1, 2]. Using the current strategy of passive case finding, the case detection rate in the region has improved from around 40% in 2002 to 65% in 2008. However, it has stagnated since 2006 and remains below the target of more than 70% [3].

A lower than expected case detection rate indicates that TB cases in the community are not being adequately identified and treated, which means ongoing transmission of TB infection [4]. The risk of transmission increases with the closeness of contact, overcrowded living conditions, and the degree of infectiousness of a TB case as determined by the positivity of sputum smear microscopy of acid-fast bacilli (AFB) and degree of lung field involvement in the chest X-ray (CXR) [5, 6]. Close contacts to a TB case such as those living in the same household are at higher risk of infection than casual contacts. Among those that are infected, young children (<5 years) or those with immunodeficiency (e.g., HIV infected) are at increased risk of developing TB disease, usually within two years following infection [7]. Therefore, the World Health Organization (WHO), the International Union against Tuberculosis and Lung Diseases (IUATLD) and the National TB Control Programs (NTPs) recommend screening of all children who are household contacts of sputum smear-positive TB case [8].

Screening and management of child contacts has great potential to reduce TB-related morbidity and mortality in children [4, 9]. It may prevent progression from infection to disease by early initiation of preventive therapy. It also can identify contacts of any age with suspected TB disease at an earlier stage than they otherwise may have presented to health care services. It, therefore, has the potential to increase case finding and reduce transmission [10]. Finally, though the contribution of young children to transmission may be small, they may form a pool of infection from which future adult cases arise [11, 12]. 

Despite the benefits, contact investigation for child contacts is rarely implemented and reported in resource-limited TB endemic settings, such as in the South East Asia. This paper aims to collate published data reporting the prevalence of TB infection and TB disease among child household contacts of TB in the South East Asia region. 

## 2. Methods

### 2.1. Search Strategies

The search strategies were developed using a combination of subject headings and keywords, including “tuberculosis,” “*Mycobacterium tuberculosis,*” “contact tracing,” “contact investigation,” “contact screening,” “household contact,” “close contact,” and “family contact.” The primary studies were searched electronically using databases PubMed, Embase, and Web of Science. Manual searching of the reference lists of the primary studies was performed to identify other eligible studies.

Published studies were included if they included children and adolescents (0–15 years), reported the yield of household contact investigation in children or provided data to calculate the prevalence of TB infection or TB disease in children, and were conducted in countries in the South East Asia region. Cross-sectional, prospective, and retrospective studies were included. The search was limited to published studies reported in English.

### 2.2. Data Extraction

Data were extracted using a modified Cochrane data extraction form. The data extracted included the following information: study site, design, description of index cases, description of household contacts, definition of household contacts, investigations performed (tuberculin skin test (TST), CXR, sputum smear microscopy of AFB, and culture of Mycobacterium tuberculosis), outcomes among child contacts (healthy, TB infection, or TB disease), and the criteria used to determine the outcomes. 

## 3. Results

The literature search revealed 1087 references of which 11 studies satisfied the inclusion criteria. There have been systematic reviews and a meta-analysis on contact investigation of TB, but none specifically assessed the yield from household contact investigation among children in the South East Asia region [13, 14].

### 3.1. Study Characteristics

Eleven eligible studies were conducted in seven countries in South East Asia: India (four studies) [15–18], Thailand (two studies) [19, 20], and one study each from Cambodia [21], Indonesia [22], Lao People's Democratic Republic [23], Pakistan [24], and Philippines [25]. Not all of the studies evaluated the prevalence of both TB infection and TB disease: five studies provided data on both; five studies evaluated the prevalence of TB infection only and one study of TB disease only. There was heterogeneity among studies with regards to epidemiology background, study design, the characteristics of the index case and child contact, and the criteria used for determining TB infection and TB disease (Table 1). 

Most studies were cross-sectional in design, and only one study conducted in India in 1960s performed a prospective followup for a period of 5 years [18]. The index case in most studies was a case of sputum smear-positive pulmonary TB (PTB). The study from Indonesia evaluated household contacts of an index case with sputum smear-negative PTB [22], and two studies in India included sputum smear-positive and smear-negative cases with abnormal CXR [15, 16]. With regard to child contacts, three studies involved children under five only [15, 21, 22], the others included older children up to 14–18 years of age [16–20, 23–25].

There was no uniform definition of a household contact across the studies, but the most common definition was a child living in the same house as the index case. Four studies specified a period of at least 3 months of living at the same house to define household contacts [19, 22, 24, 25]. A study in India defined close contact as living, cooking, and eating in the same house as the index case for the period of three months immediately preceding the start of treatment for the index case [18].

All studies which provided data on TB infection defined it as a positive TST, evaluated 48–72 after administration of tuberculin solution. However, there was a variation in the definition for a positive TST. Four studies used a cutoff of 10 mm, whereas The Philippines study used 5 mm and the Thailand study used 15 mm. Similarly, the criteria used to diagnose TB disease were different across studies. The study from Indonesia used the local scoring system [22], whereas most other studies used clinical and radiological features. 

### 3.2. The Yield of TB Infection and TB Disease among Child Contacts

The number of child contacts investigated in the studies ranged from 61 to 790 children of 50 to 342 index cases. In general, the prevalence of TB infection among child contacts under 15 years of age was higher (24.4–69.2%) than that of active TB disease (3.3–5.5%). TB disease was more commonly found among children aged less than 5 years, whereas TB infection was more common in older children (Table 2). 

**Figure 1:**
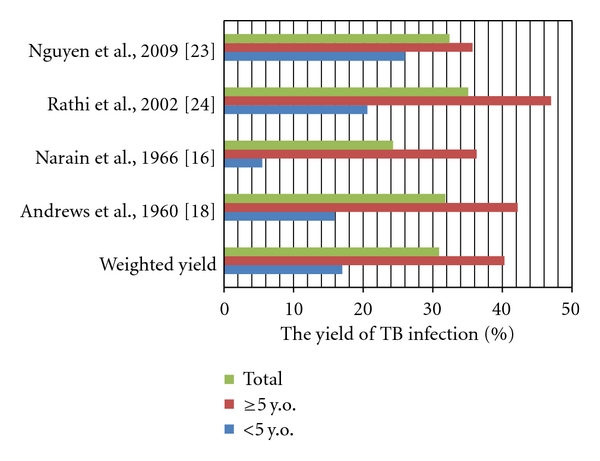
The yield of TB infection among children in household contacts.

The results of household contact investigation across the eligible studies cannot be compared directly due to the heterogeneity, particularly in outcome definitions (TB disease or TB infection).  Figure 1 presents the yield of TB infection from studies which used a cutoff of 10 mm of TST result for TB infection among sputum smear-positive index cases. It is shown that TB infection is more common in children aged more than 5 years. The prevalence of infection in all children ranged from 24.4% to 38.8%, with a weighted yield of 31%.

## 4. Discussion

The prevalence of TB infection and disease among children living in the same house as a case of pulmonary TB in South East Asia varies between settings. This variation may be due to the different epidemiology features amongst the countries or to the heterogeneity among studies with regards to the characteristics of the index case and the criteria used for determining TB infection and disease. Nonetheless, TB infection among child contacts was common and would support recommendations for routine screening and management of child household contacts. The finding that TB disease is more prevalent among young children (<5 years) is as expected given that young age is a well-established risk factor for disease following infection. 

Various approaches and criteria were used to assess the outcome of contact investigation, indicating that there has not been a universal method accepted or implemented in South East Asia. For example, the study in The Philippines used a cut-off point of 5 mm induration to define positive TST, which is lower than the recommended 10 mm in a BCG-vaccinated population [26]. The use of the cutoff may explain why this group reported the highest prevalence (67.2%) of TB infection amongst the studies. For studies which defined a positive TST as ≥10 mm induration, the proportion of TB infection ranged from 24% to 48% [6, 15, 22, 24, 27–31]. A meta-analysis of contact investigation in low- and middle-income countries by Morrison and colleagues revealed a prevalence of TB infection of 40% in children aged under 15 years [13].

Despite the evidence of high rates of infection and disease in child contacts in South East Asia, screening is rarely implemented. A cross-sectional study in India reported that only 31 of 220 (14%) children younger than 14 years living in the same house as adults with pulmonary TB were screened for TB [32]. A higher yield of 52% was reported by Tornee and co-workers in a prospective study in Thailand. [20]. The potential of contact screening upon identification of an infectious case in the community is emphasised by a recent study from South Africa which reported that most cases of young children with confirmed TB represented a missed prior opportunity for preventive therapy [33].

Isoniazid preventive therapy (IPT) which has proven efficacy in preventing TB disease in infected and uninfected child contacts is recommended for child contacts <5 years of age who have no evidence of TB disease [8, 34, 35]. Yet IPT is rarely provided. A study in India reported that, of 84 child contacts aged younger than 6 years, only 16 (19%) were initiated on IPT [36]. In Malawi, of 365 child household contacts under five, only 33 (9%) were actually screened for TB: 23 (6%) received IPT, 6 (2%) received anti-TB treatment, and, in 4 (1%), no action was taken [37]. When IPT is prescribed, poor adherence is another problem that is likely to reduce the effectiveness of this intervention [37]. Adherence rates of 15 to 28% have been reported from South Africa [38, 39].

A reason for the poor implementation of contact investigation and IPT provision in resource-limited countries is likely to be the lack of human resources. Health workers in TB endemic areas are overburdened by the identification and management of sputum smear-positive cases, which are the priority for treatment in any national TB programs. In addition, the awareness and knowledge of families and healthcare workers of the rationale and potential of IPT are lacking [33, 40]. In Malawi, only 21% of TB patients who had child contacts aged 5 years or below were informed about the need for screening their children [37]. A more recent study in Malawi in 2006 reported that only 8% of sputum smear-positive cases brought their children to the clinic for screening despite provision of clear information [41].

Guidelines that are difficult to implement may also be a barrier for the implementation of contact investigation. As long as TST and CXR remain mandatory tests for screening, coverage in resource-limited settings cannot be expected to improve, and its impact on TB control is likely to be limited. TST and CXR are not readily available in primary health clinics in the region, where index cases are commonly identified and treated. These tests are usually performed at main hospitals located in cities, which increases transport and time costs. The use and interpretation of TST are also problematic: unaffordable for most families in resource-limited setting; require skill to perform, and a second hospital visit is necessary [10]. 

A more simple, cheap, feasible, efficient and patient-friendly method is required. The current WHO guideline [8] of symptom-based screening recommends symptom evaluation alone to decide whether the contact requires further investigation for TB disease or can be prescribed IPT directly. Asymptomatic child contacts aged less than 5 years can be provided IPT without further investigation. If TB is suspected at initial assessment or at subsequent followup, further investigation should be performed to establish or exclude a diagnosis of TB disease. Referral to a district or tertiary hospital may be necessary when there are uncertainties about the diagnosis. Therefore, TST or CXR is not necessarily performed in all child contacts [8]. 

A limitation of this systematic review is that it only includes published studies reported in English. More precise yields from contact investigation among child household contacts in the South East Asia would have been gained if unpublished studies and the grey literature such as reports from NTPs of each country were included. 

## 5. Conclusion

Contact investigation studies in South East Asia indicate the potential of screening and IPT to reduce the risk of TB disease in child contacts, yet it is rarely implemented. Research is required to determine patient and health service barriers to screening to enable targeted effective intervention programs to be developed. One such research strategy could include a qualitative review of problems around the implementation of contact investigation and IPT provision for child contacts in the region. This will provide valuable information for the design of community-based interventions to improve the management of child contacts. A simple method of contact investigation standardised in the region is required.

## Figures and Tables

**Table 1:** Characteristic of the studies.

Author	Country	Year of study	Epidemiology features	Characteristics of index case	Characteristics of contact	Criteria of TST (+)	TB disease diagnosis	Method of sputum collection
Andrews et al. [18]	India	1960s	NA	SS (+)	Adults and children	At 48–72 hrs, not specified	Clinical, CXR, and/or microbiology	Laryngeal swab
Narain et al. [16]	India	1960/1961	NA	SS (+) or CXR (+)	Adults and children	Not explained	NA	No detail information for children
Kumar et al. [17]	India	1982/1983	NA	SS (+)	Adults and children	Not performed	Clinical, CXR, and/or microbiology	Spot collection
Singh et al. [15]	India	NA	NA	SS (+) and SS (−) but CXR (+)	<5 yo	10 mm at 72 hrs	Clinical and CXR	Gastric lavage
Rathi et al. [24]	Pakistan	1999	NA	>15 yo SS (+)	Adults and children	At 72 hrs: 10 mm if BCG (−) 15 mm if BCG (+)	NA	Not performed
Salazar-Vergara et al. [25]	Philippines	2001	NA	SS (+)	0–15 yo	5 mm at 48–72 hrs	Clinical, CXR, and/or microbiology	Gastric aspirate if CXR suggestive of TB
Tornee et al. [19]	Thailand	2002/2003	NA	>15 yo SS (+)	1–14 yo	15 mm at 48 hrs	NA	Not performed
Tornee et al. [20]	Thailand	2003	NA	>15 yo SS (+)	<15 yo	NA	NA	Not performed
Iskandar et al. [22]	Indonesia	2006	incidence: 245/100000 prevalence: 675/100000	Adult, SS (−)	4–60 months of age	10 mm at 72 hrs	Indonesia scoring system	Not performed
Nguyen et al. [23]	Lao	2006	incidence: 151/100000 prevalence: 289/100000	>15 y.o. SS (+)	Adults and children	10 mm at 48–72 hrs	NA	Not performed
Okada et al. [21]	Cambodia	2005	NA	Adult, SS (+) and SS (−)	<5 yo	10 mm at 72 hrs	Clinical, TST, and CXR	Not performed

NA: not available; SS (+): sputum smear positive; SS (−): sputum smear-negative; y.o.: years old; hrs: hours.

**Table 2:** The yields of household contact investigation among child contacts.

Author	Country	Number of source case	Number of child contacts	Yield (%) for TB infection			Yield (%) for TB disease		
				<5 yo	5–15 yo	<15 yo	<5 yo	5–15 yo	<15 yo

Andrews et al. [18]	India	191	398	16.2	53.6	38.8	9.2	3	5.5
Narain et al. [16]	India	75	790	5.5	36.5	24.4	NR	NR	NR
Kumar et al. [17]	India	50	142	NR	NR	NR	3.2	NR	NR
Singh et al. [15]	India	200	281	33.8	NR	NR	3.2	NR	NR
Rathi et al. [24]	Pakistan	77	151	7.8	21.8	29.6	NR	NR	NR
Salazar-Vergara et al. [25]	Philippines	62	153	51.2	76.9	69.2	NR	NR	3.3
Tornee et al. [19]	Thailand	325	480	NR	NR	47.8	NR	NR	NR
Tornee et al. [20]	Thailand	342	500	NR	NR	47.1	NR	NR	NR
Iskandar et al. [22]	Indonesia	54	61	10	NR	NR	16.4	NR	NR
Nguyen et al. [23]	Lao	72	148	26	35.7	31.1	NR	NR	NR
Okada et al. [21]	Cambodia	164	217	24	NR	NR	8.7	NR	NR

NR: not reported.
